# Deleterious Impact of a Novel *CFH* Splice Site Variant in Atypical Hemolytic Uremic Syndrome

**DOI:** 10.3389/fgene.2019.00465

**Published:** 2019-05-15

**Authors:** Ria Schönauer, Anna Seidel, Maik Grohmann, Tom H. Lindner, Carsten Bergmann, Jan Halbritter

**Affiliations:** ^1^Division of Nephrology, University Hospital Leipzig, Leipzig, Germany; ^2^Center for Human Genetics, Bioscientia, Ingelheim, Germany

**Keywords:** complement factor H, atypical hemolytic uremic syndrome, splice site variant, short consensus repeat 18, eculizumab, CFH, aHUS

## Abstract

Atypical hemolytic uremic syndrome (aHUS) is a heterogeneous disorder characterized by microangiopathic hemolytic anemia (MAHA), thrombocytopenia, and acute kidney injury (AKI). In about 50% of cases, pathogenic variants in genes involved in the innate immune response including complement factors complement factor H (CFH), CFI, CFB, C3, and membrane co-factor protein (MCP/CD46) put patients at risk for uncontrolled activation of the alternative complement pathway. As aHUS is characterized by incomplete penetrance and presence of additional triggers for disease manifestation, genetic variant interpretation is challenging and streamlined functional variant evaluation is urgently needed. Here, we report the case of a 27-year-old female without previous medical and family history who presented with confusion, petechial bleeding, and anuric AKI. Kidney biopsy revealed glomerular thrombotic microangiopathy (TMA). Targeted next generation sequencing identified a paternally transmitted novel heterozygous splice site variant in the *CFH* gene [c.3134-2A>G; p.Asp1045_Thr1053del] which resulted in a partial in-frame deletion of exon 20 transcript as determined by cDNA analysis. On the protein level, the concomitant loss of 9 amino acids in the short consensus repeat (SCR) domains 17 and 18 of CFH includes a highly conserved cysteine residue, which is assumed to be essential for proper structural folding and protein function. Treatment with steroids, plasmapheresis, and the complement inhibitor eculizumab led to complete hematological and clinical remission after several months and stable renal function up to 6 years later. In conclusion, genetic investigation for pathogenic variants and evaluation of their functional impact, in particular in the case of splice site variants, is clinically relevant and enables not only better molecular understanding but helps to guide therapy with complement inhibitors.

## Introduction

Atypical hemolytic uremic syndrome (aHUS; MIM# 235400/612922/612923/612924/615008/612925/612926) is a disease complex characterized by the uncontrolled over-activation of the alternative pathway of the complement system (AP). Clinical symptoms include microangiopathic hemolytic anemia (MAHA), thrombocytopenia, and acute kidney injury (AKI) (Maga et al., 2010). In contrast to the “typical” form of HUS initiated by infection with Shiga-toxigenic *Escherichia coli* (STEC) and characterized by diarrheal illness (D+), atypical forms (D-) have a poorer prognosis and progress to end-stage renal disease (ESRD) in 30–60% of all cases (Loirat et al., 2008; Westra et al., 2010). Pathogenic variants in AP-regulating genes including *CFH, CFI*, or *MCP*/*CD46* are found in more than 50% of aHUS-patients and variants in *CFH* represent the most frequent genetic finding (Fremeaux-Bacchi et al., 2013).

**FIGURE 1:**
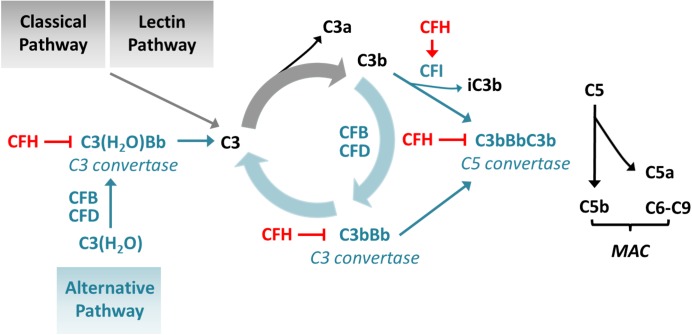
Influence of CFH on complement activation. CFH inhibits the conversion of C3 to C3b by accelerating the decay of C3 convertases and enhancing the inactivation of C3b and prevents conversion of C5 to C5b by accelerating the decay of C5 convertases. CFB: complement factor B; CFD: complement factor D; MAC: membrane attack complex.

The protein complement factor H (CFH) encoded by *CFH* plays a key role in the control of complement activation in fluid phase and on cell membranes thereby protecting self-surfaces from immune attacks. It exerts its effects by preventing the formation of the central complement protein C3b from C3 and C5b from C5 by accelerating the decay of C3 and C5 convertases (C3bBb and C3bBbC3b), respectively. Furthermore, it acts as a co-factor of complement factor I (CFI, encoded by *CFI*) in the proteolytic inactivation of C3b (Figure 1; Merinero et al., 2018).

Complement factor H is a 155 kDa plasma protein that consists of 20 homologous short consensus repeat (SCR) domains of 60 amino acids length, each containing 4 conserved cystein residues that form two structure-defining disulfide-bridges. The region spanning SCR1-4 has been proposed to act as regulatory domain, mediating C3b binding in fluid phase, CFI interaction and convertase decay accelerating activity (Goicoechea de Jorge et al., 2013; Perkins et al., 2014; Clark and Bishop, 2015; de Vriese et al., 2015; Sepúlveda et al., 2016). Whereas SCR6, SCR7, and SCR12-14 seem to be additionally involved in glycosaminoglycan (GAG) or C3b binding, SCR19 and SCR20 predominantly serve as surface-binding domain, comprising the ability to interact with both GAGs and C3b at self-membranes. Therefore, this C-terminal area is assumed to play a key role in distinguishing between host and pathogenic cells. It has been proposed that mutations within the N-terminal part of the protein often result in uncontrolled complement activation in fluid phase leading to glomerulonephritis, whereas C-terminal mutations are more frequently associated with aHUS leading to defects in the recognition and activity at the endogenous endothelia resulting in thrombotic microangiopathy (TMA) (de Vriese et al., 2015). However, the majority of pathogenic *CFH* variants show incomplete penetrance and constitute predisposing factors lowering the threshold of aHUS/TMA disease manifestation. Upon presence of additional triggers, such as infection, pregnancy, or immunosuppressive drugs, patients with predisposing variants are prone to develop aHUS/TMA. Thus, pathogenic *CFH* variants are also associated with a significant risk for disease recurrence after renal transplantation. Under these circumstances, complement-targeting therapies (e.g., eculizumab) in addition to the immunosuppressive regimen are able to provide better outcome after renal transplantation in patients with aHUS/TMA (Legendre et al., 2013; Münch et al., 2017). Therefore, genetic testing for pathogenic variants within the AP regulatory genes is indispensable to establish responsible prognosis and treatment strategies, particularly in the context of renal transplantation.

## Case Presentation

**FIGURE 2:**
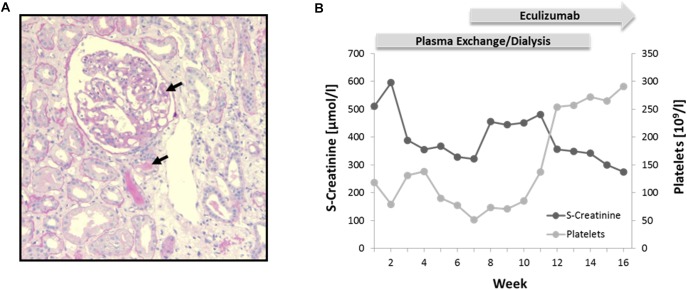
Phenotypical characterization and treatment. **(A)** Kidney biopsy showing signs of pre- and intraglomerular TMA (black arrows). Periodic Acid-Schiff (PAS) staining; 10× magnification. **(B)** Diagram illustrating course of serum (S)-creatinine and platelets during plasma exchange/dialysis and treatment with Eculizumab.

A 27-year-old female without prior medical and family history presented with nausea, confusion, petechial bleeding, and anuric AKI necessitating admission on intensive care unit and immediate initiation of renal replacement therapy by continuous veno-venous hemofiltration (CVVH). Laboratory examination revealed thrombocytopenia and Coombs-negative hemolytic anemia (hemoglobin 3.7 mmol/L, platelets 118 × 10^9^/L, haptoglobin < 0.1 g/L, fragmented erythrocytes 1%, lactate dehydrogenase > 13 mmol/L) but normal ADAMTS13 levels and activity (>50%) and absence of ADAMTS13 autoantibodies. Complement analysis yielded reduced levels for C3 (0.5 –0.7 g/l; reference range: 0.8 – 1.6) and normal levels for C4. Percutaneous kidney biopsy evidenced signs of acute and non-acute preglomerular and intraglomerular TMA (Figure 2A). The patient was initially treated with intravenous glucocorticoids and 6 weeks of plasma exchange. Only after addition of the C5-inhbitor eculizumab (4 weeks of induction followed by 6 months of maintenance) the patient’s condition slowly resolved with complete hematological and clinical remission, accompanied by gradual recovery of kidney function over several months, allowing to terminate hemodialysis (Figure 2B). Long-term follow up over 6 years showed no relapse and stable renal function at CKD stage 3 (CKD-EPI (Chronic Kidney Disease Epidemiology Collaboration) 40–50 ml/min/1.73 m^2^) without continued maintenance therapy.

**FIGURE 3:**
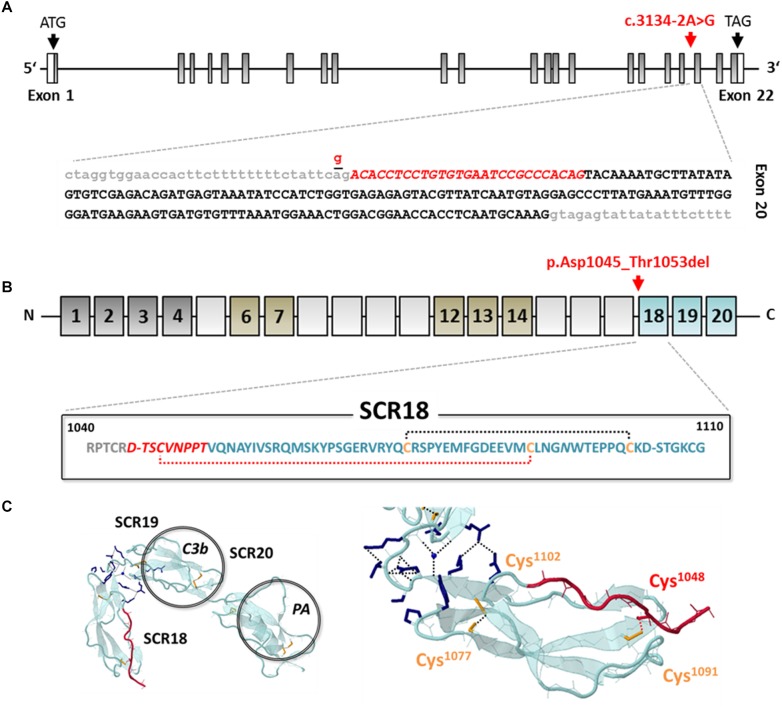
Genetic analysis and *in silico* evaluation of *CFH* c.3134-2A>G; p.Asp1045_Thr1053del. **(A)** Exon structure of the *CFH* gene indicating the position of the identified variant (red arrow and letters) at the splice acceptor site of the intron (gray letters) 19/exon (black letters) 20 boundary. The variant activates an exonic splice acceptor and leads to a partial loss of exon 20 from the RNA transcript (red italic letters). **(B)** Domain structure of CFH (dark gray: regulatory domain; brown: proteoglycan binding; blue: surface binding domain); the variant results in an in-frame deletion of 9 amino acids of SCR17 and SCR18 including Cys^1048^ (red italic letters) leading to the loss of a structure-determining disulfide bond (red-dotted line). **(C)** Model of the C-terminal domains SCR18-20 of CFH (PDB: 3SW0) modified from Morgan et al. (2012), indicating the deleted sequence part (red color) (Morgan et al., 2012). SCR, short consensus repeat; C3b, C3 binding site; PA, polyanion binding site.

Targeted next-generation sequencing using a gene panel consisting of 14 aHUS-associated genes (including *ADAMTS13, C3, CFB, CFD, CFH, CFHR1, CFHR2, CFHR3, CFHR5, CFI, DGKE, MCP, MMACHC*, and *THBD*) identified a novel heterozygous canonical splice site variant in the *CFH* gene (c.3134-2A>G; NM_000186.3), which was absent from SNP-databases (gnomAD). In addition, the following aHUS-risk alleles were detected: *CFH*-H3 (heterozygous), *MCP*-H2 (homozygous), and *CFHR1*^∗^B (homozygous). Copy number variations (CNV) of *CFH, CFHR1-3* and *CFHR5* were excluded by multiplex ligation-dependent probe amplification (MLPA). Segregation analysis yielded paternal transmission despite negative family history. The variant is located within the splice acceptor at the boundary between intron 19 and exon 20 (Figure 3A and Supplementary Figure 1). Sanger sequencing of patient cDNA indicated, that as a consequence of the variant c.3134-2A>G; r.3134_3160del, activation of an intra-exonic splice acceptor site results in an in-frame deletion of the first 27 base pairs of exon 20 transcript (Supplementary Figure 1). On the protein level, the variant leads to a loss of the very C-terminal amino acid of SCR17 and the eight N-terminal amino acids of SCR18 p.Asp1045_Thr1053del (Figure 3B). Importantly, the missing sequence includes Cys^1048^, which forms one of the two highly conserved intra-domain disulfide bonds with Cys^1091^ that is thought to be essential for correct domain folding (Figure 3C). In conclusion, this leads to a variant classification as *pathogenic* according to the American College of Medical Genetics (ACMG) (Richards et al., 2015).

Written informed consent was obtained from the participant for the publication of this report.

## Discussion

We here report a novel aHUS-associated *CFH* splice-site variant [c.3134-2A>G; p.Asp1045_Thr1053del] and predict its potential impact on the protein function by an in-frame deletion of 9 amino acids mainly affecting SCR18 of CFH. Although the activating disease trigger could not be clearly established in this case, we were able to detect the underlying genetic predisposition which allowed us to make the diagnosis of complement-mediated aHUS/TMA. In contrast to non-complement mediated forms (e.g., *DGKE, MMACHC*), patients with *CFH*-related aHUS are amenable to treatment with the C5-inhibitor eculizumab.

Complement factor H represents the key regulating protein of the alternative pathway of complement activation, required for fine-tuning of complement activity and differentiation between self-surfaces and pathogenic molecules. Consequently, the majority of pathogenic variants within the *CFH* gene lead to phenotypes related to disturbed immune processes including aHUS/TMA, C3-glomerulopathy (including dense deposit disease and C3 glomerulonephritis) or age-related macular degeneration (AMD) (Boon et al., 2008; de Córdoba and de Jorge, 2008; Morgan et al., 2012).

**FIGURE 4:**
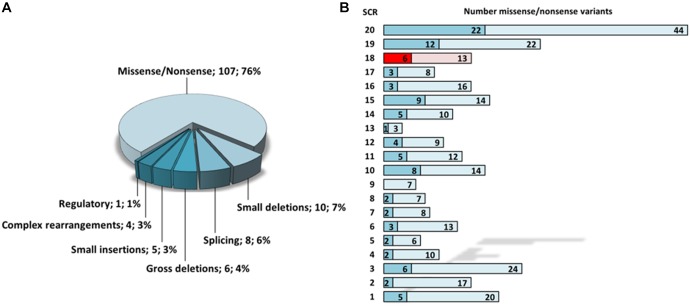
Statistical evaluation of aHUS-associated *CFH* variants according to HGMD 2018.4. **(A)** Frequency of aHUS-associated variant subtypes. **(B)** Mapping of total (light colors) and aHUS-associated (dark colors) missense/non-sense variants onto distinct SCR domains of CFH. SCR 18 is highlighted in red. SCR, short consensus repeat.

To date, a total of 346 disease-associated variants were reported for *CFH*, 141 of which refer to the phenotypical term “aHUS” (HGMD 2018.4). Of those, the vast majority (76%) represents missense or non-sense variants, whereas indels, complex rearrangements (such as *CFH*/*CFHR1* hybrid genes), and splice site variants account for less than 10% each (Figure 4A). Regarding the domain distribution of the reported missense and non-sense variants, exemplifying variants with known consequences on the protein level, the region spanning SCR19 (total = 22, aHUS-associated = 12) and SCR20 (total = 44, aHUS-associated = 22) clearly represents a mutational hotspot (Figure 4B). Thus, there is emerging evidence for the speculation that variants responsible for the aHUS phenotype might predominantly affect the C-terminal surface recognition domain (de Vriese et al., 2015; Bu et al., 2018).

To effectively allow cell protection, the CFH-molecule has to adopt a U-shaped structure that enables the interaction of its N- and C-terminal domains (SCR 1-4 and SCR19-20) with cell surface-associated C3b and GAG containing recognition sites (Morgan et al., 2012). Previously, it has been shown that SCR18 is connected to SCR19 by a flexible linker, whereas SCR19 and SCR20 are rigidly associated, allowing a kink-like conformational rearrangement of the CFH C-terminal domains with presumably functional implications. To date, six out of 13 known missense and non-sense variants and one of the variants categorized as “small deletion” [c.3269delT; p.(Met1090Serfs^∗^3)] within SCR18 were reported to be associated with aHUS. In addition, we now identified a pathogenic splice-site variant leading to the deletion of a 9 amino acid sequence mainly located within SCR18. Unfortunately, the majority of newly detected splice-site variants are not routinely subjected to further genetic or functional investigation. Thus, deeper understanding of the disease-causing effects arising from distinct transcript rearrangements remains most often elusive. For *CFH*, 13 splice-site variants have been described so far (total = IVS 2–6, 8, 16, 18, 19, 21), eight of which were associated with aHUS. Due to the domain-oriented exonic structure of the *CFH* gene, splice-site variants can affect the previous or the following SCR domain or even lead to a frame-shift resulting in a premature translational stop. The splice-site of intron 19 affected in the patient described here, was already found to be mutated in two previous reports. The variant c.3134-5T>A was found in a genetic screening of a patient cohort known to suffer from renal failure, malignant hypertension, and hypertension-associated TMA (Larsen et al., 2018). Interestingly, this exchange is located only 3bp upstream within the same splice acceptor site and *in silico* prediction tools (MutationTaster, PolyPhen2 and SIFT) resulted in mixed ratings. Additionally, the variant c.3133+1G>A that is located within the splice donor site of intron 19 was reported in a patient diagnosed with aHUS, however, only *in silico* analyses (Human Splicing Finder) were conducted suggesting abrogation of the splice donor.

By means of functional analysis, we were able to determine that the defective splice site in our case results in the activation of an intra-exonic splice acceptor, leading to the deletion of an amino acid stretch within SCR18 that contains one of the four conserved cysteine residues of the consensus sequence known to be required for the establishment of the two domain structure-defining disulfide bonds. Loss of these cysteines is highly likely to result in incorrect folding and can be assumed to severely affect protein function. 41 out of 346 total known (12%) and 23 out of 141 aHUS-associated (16%) variants (missense/non-sense) affect a cysteine residue further underlining their obvious functional importance. An accumulation of abrogated cysteine residues was also reported when considering the CFH/ membrane cofactor protein (MCP) consensus sequence (Rodriguez et al., 2014). A defective structural organization of SCR18 probably prevents the required conformational flexibility that is needed for a correct orientation of SCR19 and SCR20 which could negatively affect recognition of interaction partners and host surfaces.

## Conclusion

In summary, we detected and analyzed a novel pathogenic *CFH* splice site variant in a patient with aHUS/TMA, which probably leads to an incorrect protein fold impairing inhibitory control of AP activity. Incomplete penetrance demonstrated by the clinically asymptomatic father underlines the influence of an additional disease trigger for aHUS manifestation. Genetic assessment and functional variant analysis, particularly in the case of detected splice site variants, helps guiding patients’ treatment and assessing the probability of disease recurrence.

## Ethics Statement

This study was carried out in accordance with the recommendations of the institutional review board of the University of Leipzig with written informed consent from all subjects. All subjects gave written informed consent in accordance with the Declaration of Helsinki. The protocol was approved by institutional review board of the University of Leipzig.

## Author Contributions

RS generated and analyzed the data (genetic) and wrote the manuscript. AS generated the data (clinical) and edited the manuscript. MG generated the data (genetic) and edited the manuscript. TL contributed the clinical data. CB generated the data (genetic) and edited the manuscript. JH initiated and supervised functional evaluation, contributed clinical data, and edited the manuscript.

## Funding

JH received funding from DFG (HA 6908/2-1) and EKFS.

## Conflict of Interest Statement

MG and CB are employees of Bioscientia/Sonic Healthcare. The remaining authors declare that the research was conducted in the absence of any commercial or financial relationships that could be construed as a potential conflict of interest.

## Supplementary Material

FIGURE S1 Experimental analysis of the splice-site variant c.3134-2A>G. **(A)** Agarose gel electrophoresis showing PCR fragments of *CFH* cDNA derived from primary dermal fibroblast RNA (WT, wild type control; PAT, patient); the additional splice product is depicted with an white arrow. **(B)** Sequence of the *CFH* cDNA showing the consequences of the splice site variant; exons 18 – 20 are indicated as upper case letters (wild type sequence: alternating black and blue, missing bases: red) and intron 19 as lower case letters (gray: wildtype sequence, red: base exchange c.3134-2A>G); primer binding sites are shown in gray and sequencing results in yellow and light blue. **(C)** Chromatogram of the additional PCR-fragment (reverse sequence); assigned positions of exon 19 and exon 20 are depicted in light blue and yellow, respectively, and the missing part of exon 20 is shown in red.
