# Supplementary figures and images for: Deleterious Impact of a Novel CFH Splice Site Variant in Atypical Hemolytic Uremic Syndrome
Source: Front Genet. 2019 May 15;10:465. doi: 10.3389/fgene.2019.00465 (PMC6530248; doi:10.3389/fgene.2019.00465)

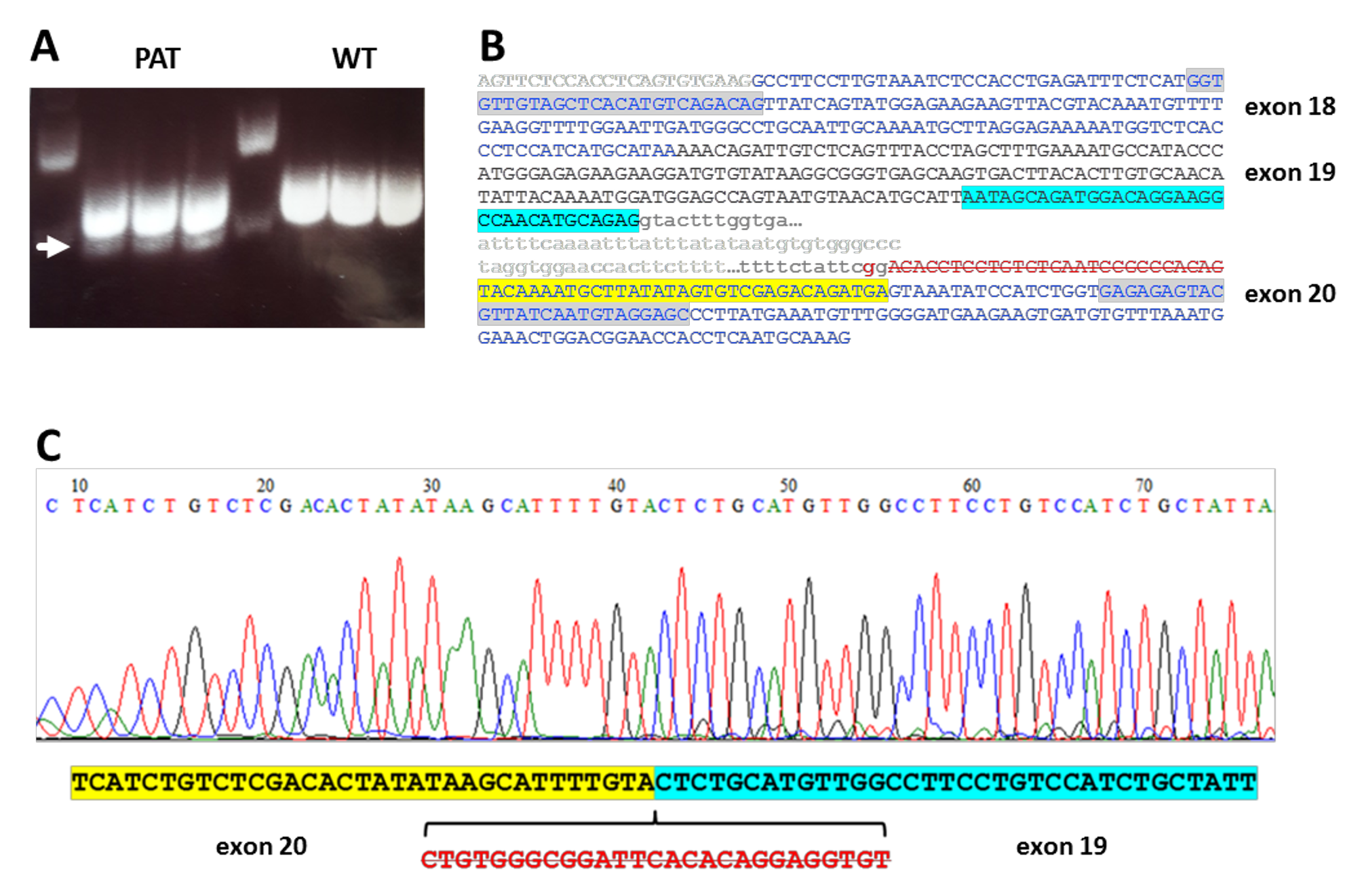

Supplement: FIGURE S1 — Experimental analysis of the splice-site variant c.3134-2A>G. (A) Agarose gel electrophoresis showing PCR fragments of CFH cDNA derived from primary dermal fibroblast RNA (WT, wild type control; PAT, patient); the additional splice product is depicted with an white arrow. (B) Sequence of the CFH cDNA showing the consequences of the splice site variant; exons 18 – 20 are indicated as upper case letters (wild type sequence: alternating black and blue, missing bases: red) and intron 19 as lower case letters (gray: wildtype sequence, red: base exchange c.3134-2A>G); primer binding sites are shown in gray and sequencing results in yellow and light blue. (C) Chromatogram of the additional PCR-fragment (reverse sequence); assigned positions of exon 19 and exon 20 are depicted in light blue and yellow, respectively, and the missing part of exon 20 is shown in red. [file Image_1.TIF]
